# Relationship between *BCL2* mutations and follicular lymphoma outcome in the chemoimmunotherapy era

**DOI:** 10.1038/s41408-023-00847-1

**Published:** 2023-05-17

**Authors:** Cristina Correia, Matthew J. Maurer, Samantha J. McDonough, Paula A. Schneider, Paige E. Ross, Anne J. Novak, Andrew L. Feldman, James R. Cerhan, Susan L. Slager, Thomas E. Witzig, Bruce W. Eckloff, Hu Li, Grzegorz S. Nowakowski, Scott H. Kaufmann

**Affiliations:** 1 Division of Oncology Research, Department of Oncology, Mayo Clinic, 200 First Street SW, Rochester, MN 55905 USA; 2 Department of Quantitative Health Sciences, Mayo Clinic, 200 First Street SW, Rochester, MN 55905 USA; 3 Medical Genome Facility, Mayo Clinic, 200 First Street, S.W., Rochester, MN 55905 USA; 4 Genomics Systems Unit, Mayo Clinic, Rochester, MN 55905 USA; 5 Division of Hematology, Department of Medicine, Mayo Clinic, 200 First Street SW, Rochester, MN 55905 USA; 6 Department of Laboratory Medicine and Pathology, Mayo Clinic, 200 First Street SW, Rochester, MN 55905 USA; 7 Department of Molecular Pharmacology and Experimental Therapeutics, Mayo Clinic, 200 First Street SW, Rochester, MN 55905 USA

**Keywords:** B-cell lymphoma, Cancer genetics

## Abstract

How to identify follicular lymphoma (FL) patients with low disease burden but high risk for early progression is unclear. Building on a prior study demonstrating the early transformation of FLs with high variant allele frequency (VAF) *BCL2* mutations at activation-induced cytidine deaminase (AICDA) sites, we examined 11 AICDA mutational targets, including *BCL2*, *BCL6*, *PAX5, PIM1*, *RHOH*, *SOCS*, and *MYC*, in 199 newly diagnosed grade 1 and 2 FLs. *BCL2* mutations with VAF ≥20% occurred in 52% of cases. Among 97 FL patients who did not initially receive rituximab-containing therapy, nonsynonymous *BCL2* mutations at VAF ≥20% were associated with increased transformation risk (HR 3.01, 95% CI 1.04–8.78, *p* = 0.043) and a trend toward shorter event-free survival (EFS, median 20 months with mutations versus 54 months without, *p* = 0.052). Other sequenced genes were less frequently mutated and did not increase the prognostic value of the panel. Across the entire population, nonsynonymous *BCL2* mutations at VAF ≥20% were associated with decreased EFS (HR 1.55, 95% CI 1.02–2.35, *p* = 0.043 after correction for FLIPI and treatment) and decreased overall survival after median 14-year follow-up (HR 1.82, 95% CI 1.05–3.17, *p* = 0.034). Thus, high VAF nonsynonymous *BCL2* mutations remain prognostic even in the chemoimmunotherapy era.

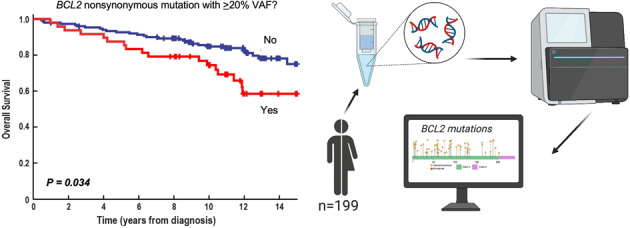

## Introduction

Follicular lymphoma (FL) is one of the most common lymphoid neoplasms, with an incidence of 10–15,000 new cases per year in the United States and a prevalence of 140,000 cases, reflecting the indolent nature of the disease in many patients [1, 2]. FL is commonly managed with observation, anti-CD20 alone (e.g., rituximab), or anti-CD20 in combination with intensive chemotherapy such as cyclophosphamide, doxorubicin, vincristine, and prednisone (CHOP) or bendamustine (BR) [1, 3–5]. Even with this chemoimmunotherapy treatment, however, ~20% of patients either do not achieve complete remission or relapse within 24 months [6, 7]. It is currently unclear how to prospectively identify those FLs at the highest risk of poor outcomes at diagnosis [8, 9].

The vast majority of FLs harbor the *t*(14;18) chromosomal translocation that juxtaposes the *BCL2* gene and the immunoglobulin heavy chain locus [10–12]. This translocation results in enhanced expression of the BCL2 protein, which inhibits apoptotic cell death [13]. In addition, the *BCL2* gene is subject to mutagenesis by activation-induced cytidine deaminase (AICDA), an enzyme concentrated at the immunoglobulin locus, where it participates in mutagenesis leading to antibody diversification in normal germinal center B cells [14, 15]. FLs have previously been reported to maintain AICDA expression [16, 17] and contain mutations in a number of AICDA-target genes, including *MYC*, *PAX5*, *SOCS1*, *BCL6*, and *RHOH*, as well as *BCL2* [18–21]. Examination by next-generation sequencing (NGS) using 1% mutant allele frequency as a cutoff for calling mutations has identified *BCL2* mutations in the vast majority of FLs and mutations in the other genes in a substantial fraction of cases [22, 23].

How to optimally manage newly diagnosed, relatively asymptomatic FL remains a topic of discussion [24], especially during the Covid-19 pandemic [25]. In the pre-rituximab era, patients with the low-volume disease were often observed until they experienced increased disease burden or FL transformation to more aggressive lymphoma. Earlier studies indicated a transformation rate of ~3%/year and a median survival of 2–3 years with intensive chemotherapy after transformation. More contemporary analysis in patients who received rituximab prior to FL transformation indicated that the rate of transformation is diminished [26]. Accordingly, newly diagnosed patients with FL and high tumor burden are typically treated with alkylator- or anthracycline-based chemoimmunotherapy. In contrast, patients with low-volume disease are treated with single-agent rituximab [27–29] or with watchful waiting until disease progression necessitates treatment [30–33], which occurs most frequently during the first five years [25]. Recent analysis continues to show that prompt treatment of early FL does not result in an overall survival benefit [25, 33], but there is a continuing need to identify patients who will do worse and could potentially benefit from earlier treatment.

With this in mind, a number of previous studies have attempted to identify clinical or biological factors that predict early progression. For example, a prognostic index termed m7-FLIPI, which consists of a group of seven genes as well as the clinical features that are part of the FL International Prognostic Index (FLIPI), has been reported to identify FLs at the highest risk of early failure after first-line chemoimmunotherapy [34]. While this prognostic index performs well in high-volume disease [34], it does not provide prognostic information in low-volume newly diagnosed FL [35, 36]. Although other parameters, such as survival to 12 or 24 months without an event, are prognostic [7, 37], it might be advantageous to determine the prognosis prior to first-line therapy rather than observing the duration of response afterward.

In a previous study, we utilized Sanger sequencing to study *BCL2* mutations in FL [38]. Our results, which had an estimated limit of detection of 20% variant allele frequency (VAF), demonstrated that (i) a subset of FLs harbored *BCL2* single nucleotide variants (SNVs) at or above that frequency, (ii) the majority of these SNVs were amino acid-altering (nonsynonymous), and (iii) presence of these prevalent *BCL2* SNVs was associated with earlier FL transformation and earlier death from lymphoma [38]. That study, however, was limited by the fact that all patients were diagnosed in the pre-rituximab era to ensure sufficient follow-up to assess prognostic significance. In contrast, two subsequent studies that examined patients who all received rituximab-containing therapy did not detect any impact of *BCL2* mutations on event-free or overall survival [34, 39], raising the possibility that rituximab might be able to mitigate any adverse impact of *BCL2* mutations.

In the present study, mutations in a broader series of AICDA-target genes have been ascertained using NGS in a group of patients diagnosed and treated after 2001, when rituximab became part of FL treatment. Results of this study not only continue to show an association between prevalent nonsynonymous *BCL2* mutations (VAF ≥20%) and early transformation of FLs treated without rituximab therapy, but also demonstrate an association between prevalent nonsynonymous *BCL2* mutations and overall survival (OS) during extended follow-up that might not be completely mitigated by early rituximab-containing therapy.

## Methods

### Study cohort and samples

This study was reviewed and approved by the Mayo Clinic Institutional Review Board. Samples were obtained from 199 patients with newly diagnosed grade 1 or 2 FL who consented to enrollment in the Molecular Epidemiology Resource (MER) of the University of Iowa/Mayo Clinic Lymphoma Specialized Program of Research Excellence (SPORE) [40] and were treated at the Mayo Clinic between 2002 and 2009. All patients had formalin-fixed paraffin-embedded (FFPE) biopsy samples available from initial diagnosis and a minimum follow-up of 5 years in 2014 when the cohort was assembled, i.e., prior to bendamustine approval. These patients were divided into groups that (i) received first-line therapy with rituximab, R-CHOP [7], or R-CVP (“Rituximab-treated”), with only three patients (1 R-mono and 2 R-CVP) receiving R maintenance; (ii) did not initially receive systemic treatment (“No systemic treatment”), i.e., were either observed or received localized radiation as their initial treatment; or (iii) received systemic treatment without rituximab (“Other”). FFPE lymph nodes from the initial diagnosis were reviewed to confirm the presence of FL and estimate tumor content (median 70%, range 50–90%). All participants were contacted every 6 months for the first three years after diagnosis and annually thereafter to determine disease progression/relapse, retreatment, and transformation. Importantly, all events, including transformation and changes in treatment as well as deaths and causes of death, were validated against medical records [40].

### DNA extraction and QC

DNA was extracted from 2–4 10 μm sections of each FFPE sample using the Qiagen QIAamp DNA FFPE kit (cat # 56404) according to the supplier’s instructions. DNA was eluted in 40 μl ATE buffer (10 mM Tris-Cl, pH 8.3, 0.1 mM EDTA, 0.04% NaN_3_), quantified using a NanoDrop 2000 UV spectrophotometer (Thermo Scientific, Wilmington, DE) and assessed for quality using Qubit and gDNA trace (Qubit BR kit, Life Technologies, Darmstadt, Germany). Approximately 10% of initially identified samples failed extraction and were replaced by additional FL samples that met the same criteria.

### Custom NGS panel and custom pool

We designed an amplicon- (~125–175 bp) based custom exome Ion Ampli Seq^TM^ Panel (Suppl. Table 1, coverage 94.3%) that is compatible with both MiSeq (Illumina) and Ion Torrent (Life Technologies) sequencing platforms. Additional custom primers (Suppl. Tables 2, 3 and ref. [38]) were designed to cover panel regions that were absent or displayed low coverage (<50×) with the AmpliSeq panel. Pools were amplified using the high-fidelity enzymes 5x Ion AmpliSeq HiFi Mix (pools 1 and 2) or the KAPA 2 G Robust HotStart DNA Polymerase (pool 3). Immediately after amplification, PCR primers were digested using FuPa reagent (Life Technologies, Carlsbad, CA, USA) and pooled.

### Library normalization and MiSeq NGS analysis

The concentrations and size distributions of the completed libraries were determined using an Agilent Bioanalyzer DNA 1000 chip (Santa Clara, CA) and Qubit fluorometry (Invitrogen, Carlsbad, CA). After quantification, each sample-specific library was normalized to 4 nM by the addition of EB buffer (Qiagen). Samples at a concentration lower than 0.5 nM were left undiluted. Forty-eight samples per NGS MiSeq run were pooled in equal volumes, loaded onto a MiSeq V.2 300 cycle reagent cartridge, and run on a MiSeq using a 2 × 150 bp paired-end configuration. Libraries were sequenced at an average coverage of 5000×.

### Controls

There were five control samples: Fresh frozen normal reference standard (Coriell NA12892), FFPE human tonsil, FFPE normal lymph node, FFPE lymphoma cell line without known *BCL2* mutations (Jeko), and an FFPE FL sample with known *BCL2* mutations. Dilution and mixing experiments were performed to assess minimal sample input and the impact of tumor heterogeneity. The properties of the sequencing panel were assessed as described in subsequent sections.

### Reproducibility

An FFPE FL sample that contained known mutations was included in each run. Using 30 ng of input DNA, the method was highly reproducible. Variant calling was also reproducible and did not vary significantly with coverage from 50× to 5000×.

### Tumor/normal admixtures

To assess the ability to detect variant alleles, the FFPE FL sample and FFPE tonsil were mixed in ratios of 1:1, 1:3, 1:7, and 1:15.

### SNV variant detection

All raw sequence data were processed from fastq files to variant calls using the tools available through the Mayo Clinic DNA sequence analysis pipeline GenomeGPS (v1.2.1). In brief, alignment was conducted with bwa-mem v0.7.12 [41] against the UCSC hg19 reference build. A custom in-house primer trimming methodology with b-trim32 [42] was used to reduce primer contamination by removing primer sequences occurring at the 5’ ends of reads. Genome Analysis ToolKit (GATK – v1.7) [43] was used to perform base quality score recalibration, indel realignment, duplicate removal, and SNP and INDEL discovery and genotyping across all samples. Variant filtering was performed using GATK VQSR Filtering. Variant calling across samples was performed in regions with a minimum sequencing depth of 20 (DP ≥20) using BEDtools v2.2.21, resulting in covering 60,301,486 bases. A consensus quality score of at least 20 (Q ≥20), as performed with vcf tools was required. Data for visualizing the differences in the amplicon coverage were extracted from the BAM files by using BEDtools [44, 45]. A fresh FFPE “normal” sample was used for FL variant calling.

### SNV variant calling

Known variants with allele frequencies ≥5% in the NCBI dbSNP (build137), BGI [46], ESP6500 (http://evs.gs.washington.edu/EVS/), and 1000 Genomes [47, 48] databases were removed from consideration. Variant annotation was conducted with the BioR variant annotation platform [49] against Ensembl v.70 and consequence prediction using SnpEffect (v.2.0.5d) and VEP (v.2.7). Presence or absence of gene mutations was reported as a binary variable. We selected silent and non-silent variants (i.e., missense, nonsense, nonstop, small insertions/deletions (InDel), splice-site, and translational start site mutations) at a variant allele frequency of ≥20%, ≥5%, or any variant allele frequency (i.e., presence of at least 20 supporting reads) for further correlation with clinical outcome.

### SNV validation

For a subset of 24 mutations that had VAF ≥20%, Sanger sequencing was performed as previously described in ref. [38] to confirm the presence of the SNV.

### Statistical analysis

The outcomes of interest were event-free survival (EFS), time to transformation (TTT), death due to lymphoma (lymphoma-specific survival, LSS), and overall survival (OS). EFS was defined as the time from diagnosis until relapse/progression, transformation, initiation of second-line therapy, or death due to any cause. EFS12 and EFS24 were defined by EFS status at 12 and 24 months from diagnosis. TTT was defined as the time from diagnosis to histologically proven transformation. LSS was defined as the time from diagnosis to death due to lymphoma, and OS was defined as the time from diagnosis to death from any cause. The relationship between *BCL2* mutation status and EFS or OS were assessed by the method of Kaplan and Meier [50]. Log-rank and Fisher’s exact tests were used to test for association between survival and categorical (age, gender, stage, and FLIPI) variables using different cut-offs for mutation prevalence (Supplemental Table 5). The Cox proportional hazards model was used to evaluate the impact of prognostic variables on OS, TTT, and LSS and to adjust for other known prognostic variables such as FLIPI (0–1, 2, and 3–5) or treatment regimen. Tests were two-sided and results were considered statistically significant if *p* < 0.05. All analyses were performed using SAS and R. The pre-study analysis plan called for examining the association between outcomes of interest and nonsynonymous *BCL2* mutations with VAF ≥20% based on the estimated limit of detection of *BCL2* mutations in our earlier study [38] and exploration of the impact of mutations in other genes using multivariate analysis.

## Results

### Mutation frequencies of *BCL2* and AICDA-target genes

To extend a prior study of *BCL2* mutations [38], we utilized targeted capture and massively parallel DNA sequencing to identify mutations in the AICDA-target genes [18–21] *BCL2*, *BCL6*, *PIM1*, *RHOH, PAX5*, *SOCS*, and *MYC* in a cohort with newly diagnosed FL. This assay was applied to initial diagnostic samples from 199 patients with grade 1 or 2 FL who were diagnosed between 2002 and 2009, a time when rituximab was available to treat FL. Initial treatment of these patients included observation (43%), single-agent rituximab (12%), chemoimmunotherapy with R-CHOP or R-CVP (34%), or some other treatment (11%). Patient characteristics and survival are summarized in Table 1. EFS and OS stratified by FLIPI for this cohort (Fig. S1) are typical of FL.

**Table 1:** Patient characteristics.

Characteristics	All (*n* = 199)	Prevalent nonsynonymous *BCL2* Mutation^a^ (*N* = 48)	All other patients (*N* = 151)	*P* value
**Age (median, range)**	61 (23–91)	61 (30–84)	61 (23–91)	0.90
**Gender, male %**	99 (50%)	30 (63%)	69 (46%)	0.043
**Stage**				
**1**	24 (12%)	3 (6%)	21 (14%)	0.36
**2**	27 (14%)	6 (13%)	21 (14%)	
**3**	51 (26%)	11 (23%)	40 (26%)	
**4**	97 (49%)	28 (58%)	69 (46%)	
**B symptoms (present)**	12(6%)	4 (8%)	8 (5%)	0.46
**LDH** > **ULN**	33 (18%)	8 (19%)	25 (18%)	0.96
**Hemoglobin** <**12**	20 (11%)	7 (16%)	13 (10%)	0.27
**Grade**				
**1–2**	175 (88%)	42 (88%)	133 (88%)	0.91
**3** **A**	24 (12%)	6 (13%)	18 (12%)	
**FLIPI**				
**0–1**	71 (36%)	10 (21%)	63 (42%)	0.032
**2**	74 (38%)	22 (46%)	50 (33%)	
**3–5**	51 (26%)	16 (33%)	38 (25%)	
**Initial therapy**				
**Non-systemic**				
**Observation**	85 (43%)	11 (23%)	74 (49%)	0.0026
**Radiation only**	12 (6%)	2 (4%)	10 (7%)	
**Rituximab containing**				
**Rituximab**		7 (15%)	16 (11%)	
**monotherapy**	23 (12%)			
**R-CVP**	33 (17%)	14 19%)	19 (13%)	
**R-CHOP**	33 (17%)	8 (17%)	25 (17%)	
**Radioimmunotherapy**	2 (1%)	2 (4%)	0	
**Other**^b^	11 (6%)	4 (8%)	7 (5%)	

^a^As indicated in the text, the cohort was dichotomized into those FLs with nonsynonymous *BCL2* mutations in Exon 2 at VAF ≥20% and all others. A breakdown of the other groups is shown in Table S4.

^b^Includes seven patients treated with CVP and three with other regimens.

Among the 199 samples initially identified, >90% yielded sufficient DNA for analysis. Samples with high-quality sequencing produced a high percentage of mapped reads (95.7%) to the reference genome, as well as mapped reads in the capture area (92.3%). Average base coverage of >5000x was observed inside the captured regions (Fig. S2).

**Fig. 1:**
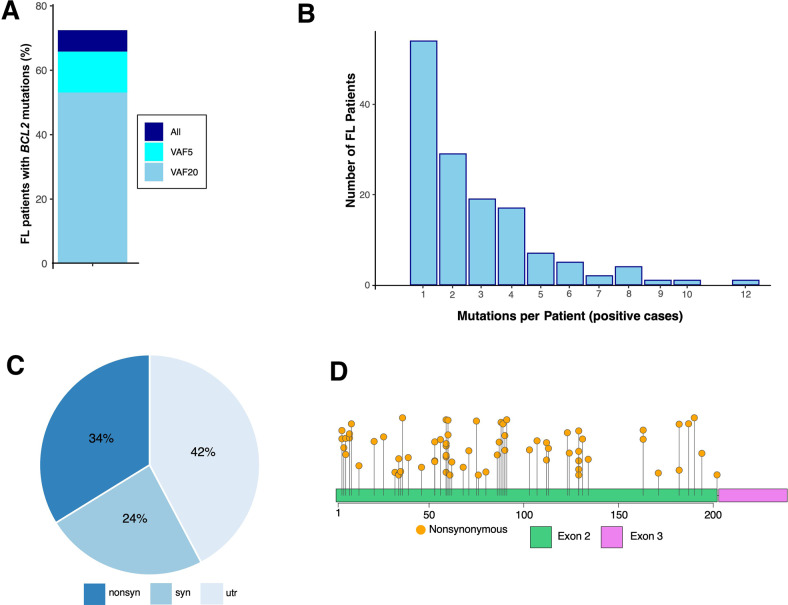
Somatic mutational profiles in FL. **A** Percentage of FL patients with somatic *BCL2* SNVs at different variant allele frequencies. **B** *BCL2* mutations per case in 103 samples harboring BCL2 mutations with VAF ≥20%. **C** Pie chart representing somatic mutation types: Synonymous, nonsynonymous (missense and nonsense), and presence in untranslated regions (urt) in 103 samples harboring *BCL2* mutations with VAF ≥20%. **D** Distribution of nonsynonymous SNVs with VAF ≥20% along the *BCL2* coding exons (2 and 3). Missense (orange) and nonsense (red) mutations are shown. An analysis showing results with VAF ≥5% is found in Supplemental Fig. S3.

Sequencing revealed that 51.8% of FLs in this cohort had *BCL2* mutations with VAF ≥20% at diagnosis (Fig. 1A). Among samples with a *BCL2* mutation, an average of 2.6 SNVs were observed in *BCL2* (range 1–12, Figs. 1B and S3A). When VAF ≥20% was used as a cutoff, these mutations were more often nonsynonymous than synonymous (Fig. 1C) and occurred predominantly in exon 2 (Fig. 1D). A similar pattern was observed when VAF ≥5% was used as a cutoff (Fig. S3B–D). The frequency of mutations in *BCL2* and other AICDA targets simultaneously was low, with only six of 103 *BCL2*-mutant FLs harboring *PIM1* mutations with VAF ≥20% (Fig. S4), a frequency that was too low to correlate with outcome in a meaningful way.

### Association between *BCL2* mutations, EFS and OS

At a median follow-up of 166 months, 133 patients (67%) had an event, 31 FLs (16%) had transformed, and 56 patients (28%) had died, 29 due to lymphoma, 11 due to other causes, and 16 due to unknown causes. The relationship between *BCL2* mutations at 20% VAF (the limit of detection of our previous study [38]) and various outcome measures such as EFS, OS, risk of transformation, and risk of lymphoma-associated death is shown in Table 2.

**Table 2:** Effects of nonsynonymous *BCL2* mutation on FL outcomes.

VAF ≥20%^a^			EFS			OS			Transformation			Lymphoma death			Non-lymphoma death		
Treatment	*N*	Adjustment	EFS	95% CI	*p* value	HR	95% CI	*p* value	HR	95% CI	*p* value	HR	95% CI	*p* value	HR	95% CI	*p* value
All	199	None	1.29	0.88–1.91	0.19	1.82	1.05–3.17	0.034	1.15	0.51–2.60	0.73	1.41	0.67–2.95	0.37	2.31	0.89–6.02	0.087
All	199	FLIPI, Treatment	1.55	1.02–2.35	0.043	1.74	0.87–3.11	0.063									
Non-systemic	97	None	1.91	0.99–3.67	0.052	2.14	0.87–5.29	0.10	3.01	1.04–8.78	0.043	1.38	0.29–6.53	0.69	2.44	0.65–9.13	0.18
Non-systemic	97	FLIPI	1.70	0.88–3.30	0.12	1.93	0.77–4.87	0.16									
R-based	91	None	1.33	0.75–2.36	0.33	1.99	0.82–4.78	0.12	0.64	0.13–3.11	0.58	1.16	0.39–3.45	0.8	N/A (3 deaths)		
R-based	91	FLIPI	1.29	0.72–2.29	0.73	1.96	0.81–4.72	0.13									

VAF ≥5%^b^			EFS			OS			Transformation			Lymphoma death			Non-lymphoma death		
Treatment	*N*	Adjustment	HR	95% CI	*p* value	HR	95% CI	*p* value	HR	95% CI	*p* value	HR	95% CI	*p* value	HR	95% CI	*p* value
All	199	None	1.15	0.80–1.65	0.44	1.63	0.96–2.78	0.073	1.03	0.48–2.19	0.94	1.07	0.52–2.20	0.85	2.54	0.99–6.53	0.053
All	199	FLIPI, Treatment	1.22	0.84–1.77	0.31	1.43	0.82–2.47	0.21									
Non-systemic	97	None	1.26	0.74–2.12	0.40	1.82	0.84–3.95	0.13	1.56	0.58–4.18	0.38	1.01	0.37–3.78	0.99	2.31	0.74–7.25	0.15
Non-systemic	97	FLIPI	1.16	0.69–1.97	0.57	1.67	0.76–3.70	0.20									
R-based	91	None	1.19	0.68–2.10	0.54	1.58	0.66–3.82	0.30	0.9	0.22–3.69	0.88	0.9	0.30–2.69	0.85	N/A (3 deaths)		
R-based	91	FLIPI	1.11	0.63–1.98	0.72	1.23	0.51–2.99	0.64									

^a^Values indicate the relationship between indicated clinical outcome and presence vs. absence of a nonsynonymous *BCL2* mutation at VAF ≥20%.

^b^Values indicate the relationship between indicated clinical outcome and the presence vs. absence of a nonsynonymous *BCL2* mutation at VAF ≥5%.

**Fig. 2:**
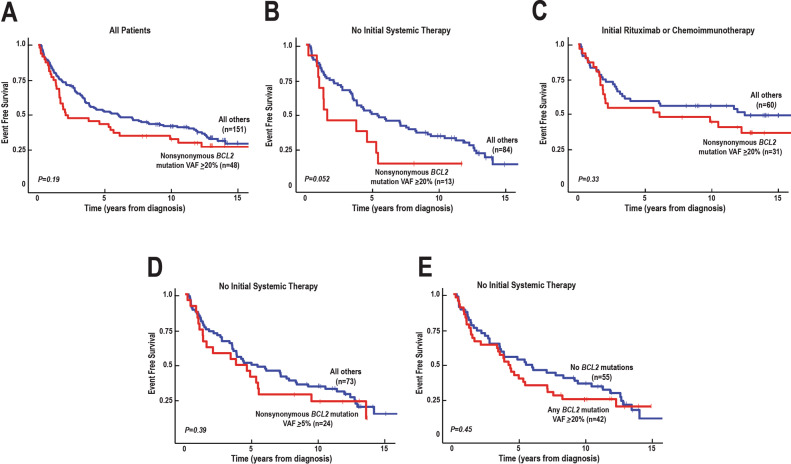
Relationship between prevalent nonsynonymous *BCL2* mutations detected at diagnosis and FL EFS. **A**–**C** Kaplan–Meier plots showing the impact of nonsynonymous *BCL2* mutations with VAF ≥20% on EFS for all patients (**A**), patients who did not receive systemic therapy initially, i.e., were observed or treated with local radiation (**B**), and those treated initially with rituximab, R-CVP or R-CHOP (**C**). **D**, **E** Kaplan–Meier plots showing the relationship between nonsynonymous *BCL2* mutations with VAF ≥5% (**D**) or any *BCL2* mutation (synonymous or nonsynonymous) with VAF ≥20% (**E**) and EFS for patients who did not receive systemic therapy initially.

In subsequent analysis, the cohort was split into those with nonsynonymous *BCL2* mutations at VAF ≥20% (the subset of patients with poor outcomes in our previous study [38], described for ease of reference as having “prevalent nonsynonymous *BCL2* mutations”) and all others. Considering all patients irrespective of treatment, the median EFS was 50 months for patients with these prevalent nonsynonymous *BCL2* mutations versus 74 months for the remaining patients (Fig. 2A, *p* = 0.19). The EFS12 was 77 vs 86% (*p* = 0.17) and EFS24 66 vs 77% (*p* = 0.19) in those with prevalent nonsynonymous *BCL2* mutations vs. those without.

When patients were grouped by initial treatment, several striking differences emerged. First, FLs with prevalent nonsynonymous *BCL2* mutations at diagnosis were less likely to be observed (11 vs 74%, *p* = 0.0026) and tended to be associated with a higher FLIPI (*p* = 0.032) (Table 1), suggesting higher risk disease. Second, in patients whose initial management did not include rituximab (i.e., those observed or treated with localized radiation), there was a trend toward lower EFS in cases with prevalent nonsynonymous *BCL2* mutations (Fig. 2B, median 20 months with these mutations vs. 54 months without, *p* = 0.052). In contrast, in patients who received rituximab as part of their initial therapy (either rituximab monotherapy or immunochemotherapy), event-free survival was greater (Fig. 2C vs. B) and, while the EFS curves separated after 2 years, the difference between FLs with prevalent nonsynonymous *BCL2* mutations and those without was not statistically significant (Fig. 2C, *p* = 0.33). Further analysis indicated that the association between nonsynonymous *BCL2* mutations and EFS in patients whose initial management did not include rituximab was only observed when prevalent (e.g., VAF ≥20%) mutations were examined and not with a lower cutoff, such as VAF ≥5% (cf., Fig. 2B vs. D). Moreover, even with 20% VAF as a cut-off, no statistically significant association was observed when all mutations were considered rather than just nonsynonymous mutations (cf., Fig. 2B vs. E).

**Fig. 3:**
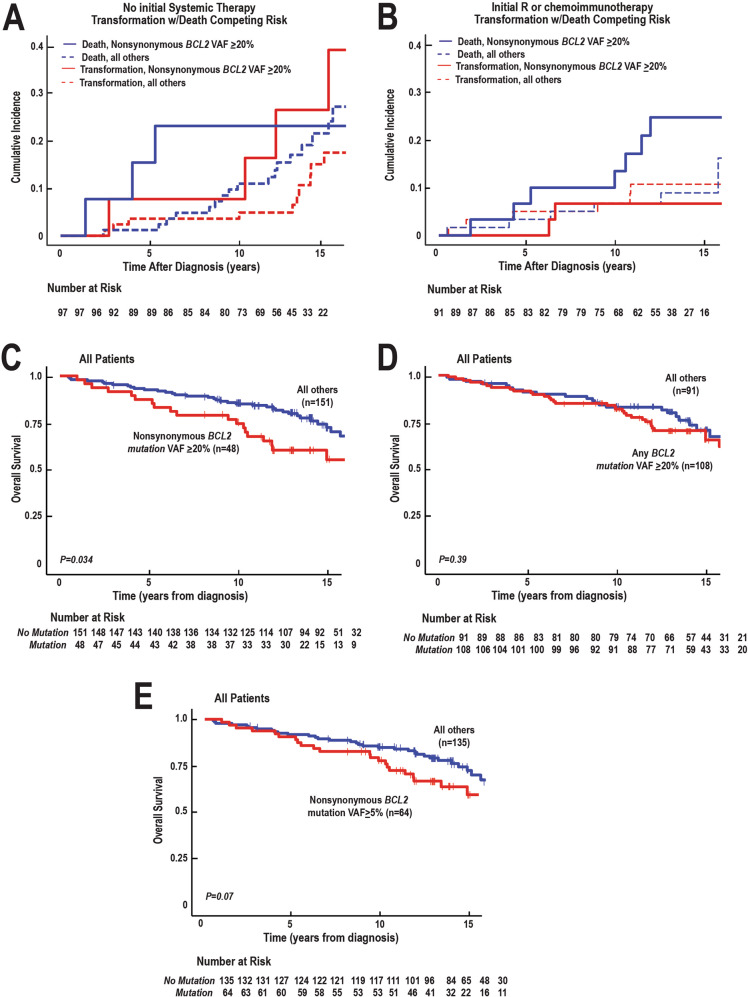
Relationship between prevalent nonsynonymous *BCL2* mutations detected at diagnosis and TTT or OS. **A**, **B** Kaplan–Meier plots showing the impact of *BCL2* nonsynonymous mutations with VAF ≥20% on TTT and time to death for patients who did not receive systemic therapy initially (**A**) or patients treated initially with rituximab, R-CVP, or R-CHOP (**B**). **C** Kaplan–Meier plot showing the impact of prevalent nonsynonymous *BCL2* mutations on OS for all FL patients. **D**, **E**, Kaplan–Meier plots showing the relationship between any *BCL2* mutation (synonymous or nonsynonymous) with VAF ≥20% (**D**) or nonsynonymous *BCL2* mutations with VAF ≥5% (**E**) and OS for all FL patients studied.

In the subset of patients who did not receive systemic treatment initially, i.e., those observed or treated only with local radiation, the presence of prevalent nonsynonymous *BCL2* mutations was also associated with earlier time to lymphoma-associated death (Fig. 3A). Conversely, in patients who received rituximab or chemoimmunotherapy at the time of initial diagnosis, the TTT was indistinguishable between FLs with prevalent nonsynonymous *BCL2* mutations and those without (Fig. 3B). Nonetheless, beyond 10 years the rate of lymphoma-associated death even in this rituximab-treated subgroup increased if prevalent nonsynonymous *BCL2* mutations were present at diagnosis (Figs. 3B and S5). Accordingly, the OS across the entire cohort was significantly shorter for FLs that harbored nonsynonymous *BCL2* mutations compared with those that did not (*p* = 0.034, Fig. 3C). In contrast, when all *BCL2* mutations are considered (both synonymous and nonsynonymous), there was no significant association between VAF ≥20% *BCL2* mutations and overall survival (Fig. 3D). Moreover, while the trend remained, the association between nonsynonymous *BCL2* mutations and overall survival was no longer significant when a VAF of ≥5% was used as a cutoff (Fig. 3E).

## Discussion

Despite advances in FL diagnosis and therapy, there is still a pressing need to identify patients whose disease is likely to progress rapidly so that alternative treatments can be considered. Here we developed an NGS assay that examined common mutational targets in FL. Looking across a number of AICDA targets, the majority of high VAF SNVs were detected in *BCL2*, in agreement with other studies [34, 51]. Further analysis showed that the presence of VAF ≥20% nonsynonymous *BCL2* mutations was associated with an increased risk of transformation in patients who initially received no systemic therapy as well as evidence of decreased OS across the entire FL cohort. These observations have potential implications for current FL management strategies.

For this study, we examined 199 patients with newly diagnosed grade 1 or 2 FL treated at a single institution between 2002 and 2009. In this cohort, patients were divided into those who initially received rituximab with or without chemotherapy versus those who initially received no systemic therapy (observed or treated with local therapy). These patients were derived from the MER, a previously described database of lymphoma patients [7, 40, 52, 53], and were specifically chosen to have a minimum of 12 years of follow-up at the time of the present analysis.

Mutations in *BCL2* at an allele frequency of >1% were detectable in 72.5% of these patients at diagnosis. Moreover, *BCL2* mutations in allele frequencies of 5 or 20% were observed in 65.3 and 52% of these FLs, respectively. As in previous studies [38, 39], most of these SNVs occurred at Cs and Gs; over half of the mutations in the coding region were nonsynonymous; and the vast majority were detected in exon 2 (Fig. 1C, D and S3). Previous analysis has shown that many of these nonsynonymous SNVs represent *BCL2* gain-of-function mutations [38].

When the presence of these mutations was examined in the context of clinical outcomes in the chemoimmunotherapy era (Table 2), several interesting patterns emerged. First, among patients who initially were treated without rituximab (i.e., with watchful waiting or only localized radiation), there was an increased risk of transformation of FLs with VAF ≥20% nonsynonymous *BCL2* mutations compared to FLs without these prevalent nonsynonymous *BCL2* mutations (Fig. 3A, HR 3.01, 95% CI 1.04–8.78) in agreement with our previous results obtained in an earlier, non-overlapping cohort treated without rituximab [38].

Second, the association of VAF ≥20% nonsynonymous *BCL2* mutations with FL transformation varied depending on whether patients received initial rituximab-containing therapy or not. In particular, patients who received rituximab-containing therapy had a lower risk of FL transformation than those who did not (cf., Fig. 3A, B), in agreement with earlier studies reporting a lower transformation rate in patients receiving rituximab-containing therapy [26, 53]. Moreover, there was no detectable deleterious effect of nonsynonymous VAF ≥20% *BCL2* mutations on transformation in FL patients who initially received rituximab-containing therapy (Fig. 3B).

Third, the presence of VAF ≥20% nonsynonymous *BCL2* mutations appears to be associated with worse OS across the entire population (Fig. 3C). While this might seem to be at odds with the similar EFS of the overall population regardless of whether FLs contain or lack prevalent nonsynonymous *BCL2* mutations (Fig. 2A), it is important to emphasize that FL is an indolent disease that often is treated with multiple regimens after the initial event occurs. Even in patients initially treated with rituximab, the present results suggest an increased rate of lymphoma-related death beyond 10 years if prevalent nonsynonymous *BCL2* mutations are present at diagnosis (Fig. 3B, blue solid line), raising the possibility that increased drug resistance in later-line therapy might provide an explanation for the separation between the two survival curves in Fig. 3C.

When combined with our prior study [38], the present results suggest that high VAF nonsynonymous *BCL2* mutations might be associated with increased FL transformation and increased lymphoma-associated death. This impact appears to be particularly strong in patients who do not receive rituximab as part of their initial therapy, i.e., are observed or treated with localized therapy (Fig. 3A). Because of the number of different sequential treatments that FL patients receive during the course of their disease, it is very unusual for any genomic feature in FL at diagnosis to have an impact on survival.

One potential implication of these observations is that sequencing of *BCL2* for VAF ≥20% nonsynonymous mutations might complement other FL prognostic tests. In particular, as indicated in the Introduction, assessment of EFS at 12 or 24 months is prognostic but cannot be determined at diagnosis. The m7-FLIPI score can be assessed at diagnosis but does not perform well in low-volume disease. In contrast, the presence of nonsynonymous *BCL2* mutations at VAF ≥20% can be assessed at diagnosis, identifies a subset of patients at risk for early transformation and death if they do not receive initial rituximab-containing therapy (Fig. 3A), and provides information about OS in the FL population (Fig. 3C).

Our suggestion that *BCL2* mutations might impact survival in FL appears to be at odds with other recent studies [34, 39]. Importantly, however, the present work differs from these other studies in several potentially important ways. First, rather than count any *BCL2* mutation, no matter how low in abundance, we focused on nonsynonymous SNVs with VAF ≥20%, a cut-off chosen to approximate the limit of detection in our earlier study [38]. Second, our study had more prolonged follow-up than other studies. With a follow-up of ~7 years, Huet et al. observed persistent separation of the survival curves based on *BCL2* mutation status in patients from the PRIMA study, but this did not reach statistical significance [39]. In contrast, the present study followed patients for up to 16 years. Given the possibility that prevalent nonsynonymous *BCL2* mutations might be associated at points beyond 10 years with an increased lymphoma-associated death rate in patients receiving initial rituximab-containing therapy (Fig. 3B), the length of follow-up might be an important difference between the studies.

Keeping in mind that the nonsynonymous *BCL2* mutations have been shown, at least in some cases, to be gain-of-function mutations that impair the induction of apoptosis [38], it is interesting to speculate how rituximab might partially mitigate the effect of these mutations. Although rituximab-induced apoptosis has been reported to occur through the BCL2 suppressible mitochondrial apoptotic pathway [54], rituximab also has been reported to activate antibody-dependent cytotoxicity [1, 55], which is likely *BCL2*-independent. To the extent that anti-CD20 antibodies kill FL cells through a BCL2-insensitive pathway, they would be expected to blunt the adverse effects of gain-of-function *BCL2* mutations. On the other hand, the presence of *BCL2* mutations also provides the opportunity to further investigate the ability of alternative therapies, such as the dendrimer-conjugated BCL2/BCLX_L_ inhibitor AZD0466 [56], to overcome the impact of *BCL2* mutations.

Despite its strengths, the present study also has several potential limitations. First, while the present results suggest many conclusions similar to those reported after the study of samples from the pre-rituximab era [38], the present study does not have a second cohort for validation of the current methodology. Second, treatment was not uniform, reflecting the fact that the present patients were not part of a clinical trial. Although this is a limitation, the treatments described here reflected practice in the hematology community and provided the opportunity to compare the impact of prevalent nonsynonymous *BCL2* mutations in FLs that were initially observed versus those initially treated with rituximab-containing therapy. Third, the present study focused on the initial therapy and did not model second or later-line therapies. Fourth, even though samples for the present study were accrued after the use of rituximab became widespread, the study did not include samples from patients receiving more recently introduced agents such as bendamustine or obinutuzumab in order to see that the patients had a long follow-up. Fifth, the present sample set is relatively small. As a result, we cannot, for example, rule out the possibility that there is a small effect of prevalent nonsynonymous *BCL2* mutations on TTT, even in the rituximab-treated subset. Finally, the underlying mechanistic basis for these observations requires further evaluation. Based on the observation that variants must be both nonsynonymous and prevalent for the associations to be observed (Figs. 2, 3), it is tempting to speculate that FLs with higher *BCL2* VAFs harbor variants that afford better protection against apoptosis during lymphomagenesis. Initial work to address the anti-apoptotic effects of these variants, however, did not examine enough SNVs to allow assessment of this possibility [38]. These limitations provide an argument for further study of *BCL2* mutations in FL.

If further studies continue to suggest an association between prevalent nonsynonymous *BCL2* mutations and clinical outcomes in FL, this might have several potential implications for future FL management. First, for patients who are contemplating watchful waiting or localized therapy after FL initial diagnosis, *BCL2* sequencing might be useful to help guide treatment choice. Second, for clinical trials enrolling patients with newly diagnosed FL, stratification of patients based on *BCL2* mutation status might help assure that outcomes are not confounded by the association between *BCL2* mutation status and early progression. Finally, during the development of therapies targeting *BCL2* in novel ways, it might be informative to assess whether a particular benefit is seen in the subset of FL patients with prevalent nonsynonymous *BCL2* mutations.

## Supplementary information

Supplemental Material

## Data Availability

BAM files and clinical metadata are available from the dbGAP database under accession dbGaP accession phs002845.v1.p1.

## References

[1] Carbone A, Roulland S, Gloghini A, Younes A, von Keudell G, Lopez-Guillermo A (2019). Follicular lymphoma. Nat Rev Dis Prim.

[2] Cerhan JR (2020). Epidemiology of follicular lymphoma. Hematol Oncol Clin North Am.

[3] Leonard JP, Nastoupil LJ, Flowers CR (2018). Where to start? Upfront therapy for follicular lymphoma in 2018. Hematol Am Soc Hematol Educ Program.

[4] Kahl B (2021). High-risk follicular lymphoma: treatment options. Hematol Oncol.

[5] Cahill KE, Smith SM (2022). Follicular lymphoma: a focus on current and emerging therapies. Oncology.

[6] Casulo C, Byrtek M, Dawson KL, Zhou X, Farber CM, Flowers CR (2015). Early relapse of follicular lymphoma after rituximab plus cyclophosphamide, doxorubicin, vincristine, and prednisone defines patients at high risk for death: an analysis from the National LymphoCare Study. J Clin Oncol.

[7] Maurer MJ, Bachy E, Ghesquieres H, Ansell SM, Nowakowski GS, Thompson CA (2016). Early event status informs subsequent outcome in newly diagnosed follicular lymphoma. Am J Hematol.

[8] Casulo C (2021). Follicular lymphoma: is there an optimal way to define risk?. Hematol Am Soc Hematol Educ Program.

[9] Mozas P, Rivero A, Lopez-Guillermo A (2021). Past, present and future of prognostic scores in follicular lymphoma. Blood Rev.

[10] Tsujimoto Y, Cossman J, Jaffe E, Croce C (1985). Involvement of the bcl-2 gene in human follicular lymphoma. Science.

[11] Kumar E, Pickard L, Okosun J (2021). Pathogenesis of follicular lymphoma: genetics to the microenvironment to clinical translation. Br J Haematol.

[12] Gordon MJ, Smith MR, Nastoupil LJ. Follicular lymphoma: the long and winding road leading to your cure? Blood Rev. 2022;57:100992. 10.1016/j.blre.2022.100992 35908982

[13] Vaux DL, Cory S, Adams JM (1988). Bcl-2 gene promotes haemopoietic cell survival and cooperates with c-myc to immortalize pre-B cells. Nature..

[14] Chandra V, Bortnick A, Murre C (2015). AID targeting: old mysteries and new challenges. Trends Immunol.

[15] Casellas R, Basu U, Yewdell WT, Chaudhuri J, Robbiani DF, Di Noia JM (2016). Mutations, kataegis and translocations in B cells: understanding AID promiscuous activity. Nat Rev Immunol.

[16] Smit LA, Bende RJ, Aten J, Guikema JE, Aarts WM, van Noesel CJ (2003). Expression of activation-induced cytidine deaminase is confined to B-cell non-Hodgkin’s lymphomas of germinal-center phenotype. Cancer Res.

[17] Hardianti MS, Tatsumi E, Syampurnawati M, Furuta K, Saigo K, Nakamachi Y (2004). Activation-induced cytidine deaminase expression in follicular lymphoma: association between AID expression and ongoing mutation in FL. Leukemia.

[18] Bodor C, Bognar A, Reiniger L, Szepesi A, Toth E, Kopper L (2005). Aberrant somatic hypermutation and expression of activation-induced cytidine deaminase mRNA in mediastinal large B-cell lymphoma. Br J Haematol.

[19] Halldorsdottir AM, Fruhwirth M, Deutsch A, Aigelsreiter A, Beham-Schmid C, Agnarsson BA (2008). Quantifying the role of aberrant somatic hypermutation in transformation of follicular lymphoma. Leuk Res.

[20] Iqbal J, Naushad H, Bi C, Yu J, Bouska A, Rohr J (2016). Genomic signatures in B-cell lymphoma: how can these improve precision in diagnosis and inform prognosis?. Blood Rev.

[21] Huet S, Sujobert P, Salles G (2018). From genetics to the clinic: a translational perspective on follicular lymphoma. Nat Rev Cancer.

[22] Pasqualucci L, Khiabanian H, Fangazio M, Vasishtha M, Messina M, Holmes AB (2014). Genetics of follicular lymphoma transformation. Cell Rep..

[23] Okosun J, Bodor C, Wang J, Araf S, Yang CY, Pan C (2014). Integrated genomic analysis identifies recurrent mutations and evolution patterns driving the initiation and progression of follicular lymphoma. Nat Genet.

[24] Cartron G, Trotman J (2022). Time for an individualized approach to first-line management of follicular lymphoma. Haematologica..

[25] Arushi K, Mwangi R, Ansell SM, Habermann TM, Cerhan JR, Strouse C (2021). Patterns of therapy initiation during the first decade for patients with follicular lymphoma who were observed at diagnosis in the rituximab era. Blood Cancer J.

[26] Federico M, Caballero Barrigon MD, Marcheselli L, Tarantino V, Manni M, Sarkozy C (2018). Rituximab and the risk of transformation of follicular lymphoma: a retrospective pooled analysis. Lancet Haematol.

[27] Ardeshna KM, Qian W, Smith P, Braganca N, Lowry L, Patrick P (2014). Rituximab versus a watch-and-wait approach in patients with advanced-stage, asymptomatic, non-bulky follicular lymphoma: an open-label randomised phase 3 trial. Lancet Oncol.

[28] Kahl BS, Hong F, Williams ME, Gascoyne RD, Wagner LI, Krauss JC (2014). Rituximab extended schedule or re-treatment trial for low-tumor burden follicular lymphoma: eastern cooperative oncology group protocol e4402. J Clin Oncol.

[29] Kahl BS, Yang DT (2016). Follicular lymphoma: evolving therapeutic strategies. Blood..

[30] Horning SJ, Rosenberg SA (1984). The natural history of initially untreated low-grade non-Hodgkin’s lymphomas. N Engl J Med.

[31] Kahl B (2012). Is there a role for “watch and wait” in follicular lymphoma in the rituximab era?. Hematol Am Soc Hematol Educ Program.

[32] Ansell SM (2014). Follicular lymphoma: watch and wait is watch and worry. Lancet Oncol.

[33] Cencini E, Fabbri A, Mecacci B, Bocchia M (2020). How to manage early-stage follicular lymphoma. Expert Rev Hematol.

[34] Pastore A, Jurinovic V, Kridel R, Hoster E, Staiger AM, Szczepanowski M (2015). Integration of gene mutations in risk prognostication for patients receiving first-line immunochemotherapy for follicular lymphoma: a retrospective analysis of a prospective clinical trial and validation in a population-based registry. Lancet Oncol.

[35] Lockmer S, Ren W, Brodtkorb M, Ostenstad B, Wahlin BE, Pan-Hammarstrom Q (2020). M7-FLIPI is not prognostic in follicular lymphoma patients with first-line rituximab chemo-free therapy. Br J Haematol.

[36] Jurinovic V, Passerini V, Oestergaard MZ, Knapp A, Mundt K, Araf S (2019). Evaluation of the m7-FLIPI in patients with follicular lymphoma treated within the gallium trial: EZH2 mutation status may be a predictive marker for differential efficacy of chemotherapy. Blood..

[37] Casulo C, Dixon JG, Le-Rademacher J, Hoster E, Hochster HS, Hiddemann W (2022). Validation of POD24 as a robust early clinical end point of poor survival in FL from 5225 patients on 13 clinical trials. Blood..

[38] Correia C, Schneider PA, Dai H, Dogan A, Maurer MJ, Church AK (2015). BCL2 mutations are associated with increased risk of transformation and shortened survival in follicular lymphoma. Blood..

[39] Huet S, Szafer-Glusman E, Tesson B, Xerri L, Fairbrother WJ, Mukhyala K (2017). BCL2 mutations do not confer adverse prognosis in follicular lymphoma patients treated with rituximab. Am J Hematol.

[40] Cerhan JR, Link BK, Habermann TM, Maurer MJ, Feldman AL, Syrbu SI (2017). Cohort profile: the lymphoma specialized program of research excellence (SPORE) molecular epidemiology resource (MER) cohort study. Int J Epidemiol.

[41] Li H, Durbin R (2009). Fast and accurate short read alignment with Burrows-Wheeler transform. Bioinformatics..

[42] Kong Y (2011). Btrim: a fast, lightweight adapter and quality trimming program for next-generation sequencing technologies. Genomics..

[43] McKenna A, Hanna M, Banks E, Sivachenko A, Cibulskis K, Kernytsky A (2010). The Genome Analysis Toolkit: a MapReduce framework for analyzing next-generation DNA sequencing data. Genome Res.

[44] Quinlan AR, Hall IM (2010). BEDTools: a flexible suite of utilities for comparing genomic features. Bioinformatics..

[45] Quinlan AR (2014). BEDTools: the Swiss-Army Tool for genome feature analysis. Curr Protoc Bioinforma.

[46] Li Y, Vinckenbosch N, Tian G, Huerta-Sanchez E, Jiang T, Jiang H (2010). Resequencing of 200 human exomes identifies an excess of low-frequency non-synonymous coding variants. Nat Genet.

[47] Genomes Project C, Abecasis GR, Altshuler D, Auton A, Brooks LD, Durbin RM (2010). A map of human genome variation from population-scale sequencing. Nature..

[48] Genomes Project C, Abecasis GR, Auton A, Brooks LD, DePristo MA, Durbin RM (2012). An integrated map of genetic variation from 1,092 human genomes. Nature..

[49] Kocher JP, Quest DJ, Duffy P, Meiners MA, Moore RM, Rider D (2014). The biological reference repository (BioR): a rapid and flexible system for genomics annotation. Bioinformatics..

[50] Kaplan E, Meier P (1958). Nonparametric estimation from incomplete observations. J Am Stat Assoc.

[51] Kridel R, Chan FC, Mottok A, Boyle M, Farinha P, Tan K (2016). Histological transformation and progression in follicular lymphoma: a clonal evolution study. PLoS Med.

[52] Nowakowski GS, Maurer MJ, Habermann TM, Ansell SM, Macon WR, Ristow KM (2010). Statin use and prognosis in patients with diffuse large B-cell lymphoma and follicular lymphoma in the rituximab era. J Clin Oncol.

[53] Link BK, Maurer MJ, Nowakowski GS, Ansell SM, Macon WR, Syrbu SI (2013). Rates and outcomes of follicular lymphoma transformation in the immunochemotherapy era: a report from the University of Iowa/MayoClinic Specialized Program of Research Excellence Molecular Epidemiology Resource. J Clin Oncol.

[54] Eeva J, Nuutinen U, Ropponen A, Matto M, Eray M, Pellinen R (2009). The involvement of mitochondria and the caspase-9 activation pathway in rituximab-induced apoptosis in FL cells. Apoptosis.

[55] Pierpont TM, Limper CB, Richards KL (2018). Past, Present, and Future of Rituximab-The World’s First Oncology Monoclonal Antibody Therapy. Front Oncol.

[56] Patterson CM, Balachander SB, Grant I, Pop-Damkov P, Kelly B, McCoull W (2021). Design and optimisation of dendrimer-conjugated Bcl-2/xL inhibitor, AZD0466, with improved therapeutic index for cancer therapy. Commun Biol.

