# Supplementary material for: Relationship between BCL2 mutations and follicular lymphoma outcome in the chemoimmunotherapy era
Source: Blood Cancer J. 2023 May 17;13(1):81. doi: 10.1038/s41408-023-00847-1 (PMC10188323; doi:10.1038/s41408-023-00847-1)
Supplement: Supplementary file 1 — Supplemental Material [file 41408_2023_847_MOESM1_ESM.pdf]

Supplemental Material to

Relationship Between *BCL2* Mutations and Follicular Lymphoma Outcome in the Chemoimmunotherapy Era

Cristina Correia, Matthew J. Maurer, Samantha J. McDonough, Paula A Schneider,
Paige E. Ross, Anne J. Novak, Andrew L. Feldman, James R. Cerhan, Susan L. Slager,
Thomas E. Witzig, Bruce W. Eckloff, Hu Li, Grzegorz S. Nowakowski, and Scott H. Kaufmann

Supplemental Table 1
Ion AmpliSeq sequencing custom panel specifications for 9 genes in FL

	Coverage	Amplicons (number)	Amplicon size range (bp)*	Primer Pools (number)	Missed region (bp)	Panel size (kb)	DNA input Recommendation (per pool)
FL	94.3	165	125 - 175	2	835	19.03	10 ng

*Amplicon size ranges are quoted as length with primer sequences.

Supplemental Table 2 Sequencing custom amplicon coverage in FL

Gene	Coverage* (%)	Missing regions (supplemented with custom primers)
BCL2	95.0	yes
BCL6	87.4	yes
BCL6 untranslated	87.4	-
BIM	94.4	yes
FAS	88.7	-
PIM1	96.9	yes
PAX5	96.9	-
MYC	90.9	yes
RHOH	89.0	-
SOCS1	84.3	-
**Total**	94.3	

Summary of captured targeted genes. Genes were selected based on the presence of recurrent somatic mutations and genes subjected to SHM in FL.

* In the Ion Amp seq design there were coverage gaps across coding sequences of *BCL2, BCL6, BIM (BCL2L11), PIM1, MYC*. We designed custom primers for regions that displayed low coverage (<50x) in the AmpliSeq™ pool (See Supplemental Table 3). Samples were supplemented with pool 3 to overcome this limitation and allow for ~100% coverage.

Supplemental Table 3
Custom amplicon primers

Gene	Forward Primer	Reverse Primer
BCL2	CCGGTTGGGATTCCTGCGGATTGAC	GTAAGTTCTCTGCACAGG
BCL2	GTCCAGCCAGCTGCACCTGACGCC	TTCAGGTACTCAGTCATCCACAGGGC
BCL6	GGCAGCGGTCACACTTGTAGG	ACCCTCCAACAGAGAACGGGG
BCL6	GCCTCCCGGAGTTACCCAGAA	CGTACGGCTTGTGATCTCTCT
BIM	CAGCTGGAACTCAGGGAGGAT	CCAACATGGTGAAACCCATCTCT
BIM	GTGACAGTCTCACTCTGTCACC	
MYC	AGGCCACAGCAAACCTCCTCA	CTGATCTGTCTCAGGACTCTG
PIM1	CGCAGCCACAGCCGCAACGCCAC	GGCGCAGGGACGGTGCCCAGAGG
PIM1	TAACGCGGCCCCCTCGCCCCTGCA	GCACCGGCTCGGGCCTCTCCA
SOCS1	CGGGCTCGGCGCGCAGCCGCT	TCCGTTCGCACGCCGATTACC
SOCS1	AGGTTCTGGCCGCCGTCGGGG	GCTCACCTCTTTGTCTCTCCC
RHOH	GCCCACAGTGTACGAGAACACAG	CAGCACCACGTCTGCCTGCTGGTAG

Supplemental Table 4

Frequency of BCL2 mutations in various categories

Mutation Status	Non Synonymous	VAF20 exon2	NonSynonymous VAF20 exon2	Frequency
NO	NO	NO	NO	28%
YES	NO	NO	NO	15%
YES	NO	YES	NO	18%
YES	YES	NO	NO	12%
YES	YES	YES	NO	3%
YES	YES	YES	YES	24%

Supplemental Table 5
Relationship Between Prevalence of Somatic *BCL2* Mutations and Clinical Features

		5 % VAF		5 % VAF non-syn		20 % VAF		20 % VAF Exon2		20 % VAF non-syn	
**Characteristic**	**N**	**n (%)**	**P-value**	**n (%)**	**P-value**	**n (%)**	**P-value**	**n (%)**	**P-value**	**n (%)**	**P-value**
**Age <60**	98	61 (62%)	0.032*	29 (30%)	0.44	51 (52%)	0.53	44 (45%)	0.71	24 (24%)	0.9
**Age >60**	101	77 (76%)		35 (35%)		57 (56%)		48 (48%)		24 (24%)	
**Female**	100	68 (68%)		27 (27%)		56 (56%)		46 (46%)		18(18%)	
**Male**	99	70 (71%)	0.68	37 (37%)	0.12	52 (53%)	0.62	46 (46%)	0.95	30 (30%)	0.043*
**Stage I-II**	48	28 (58%)	0.074	10 (21%)	0.072	18 (38%)	0.011*	16 (33%)	0.049*	7 (15%)	0.11
**Stage III-IV**	147	106 (72%)		51 (35%)				73 (50%)			
**< 5 Nodal**	120	77 (64%)	0.046*	30 (35%)	0.071	58 (48%)	0.042*	50 (42%)	0.13	22 (18%)	0.02*
**5+ Nodal**	76	59 (78%)		33 (43%)		48 (63%)		40 (53%)		25 (33%)	
**FLIPI 0-1**	70	40 (57%)	0.0082**	16 (23%)	0.049*	26 (37%)	0.0006**	23 (33%)	0.0085**	10 (14%)	0.024*
**FLIPI 2-5**	126	96 (75%)		46 (37%)		79 (63%)		66 (52%)		36 (29%)	
**Observation**	85	53 (62%)	0.056	22 (26%)	0.11	37 (44%)	0.081	30 (35%)	0.0075**	11 (13%)	0.0017**
**Treated**	114	84 (75%)		41 (37%)		70 (63%)		61 (55%)		36 (32%)	

p values were calculated by χ^2 tests. * and ** indicate p <0.05 and p <0.01, respectively.

SUPPLEMENTAL FIGURES

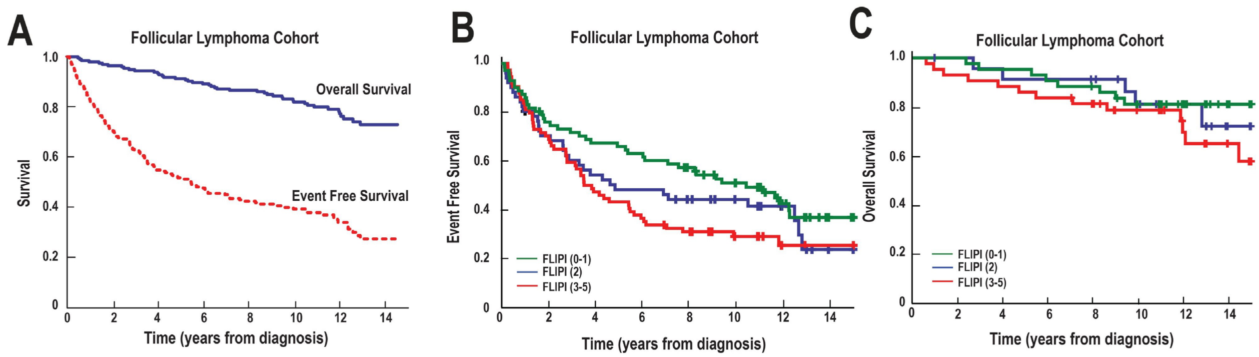

Figure S1. Summary of survival in the FL cohort. A Overall survival and event free survival of entire study population. **B, C** Event free survival **(B)** and overall survival **(C)** in the cohort stratified by FLIPI as low (0-1), moderate (2) and high risk (3-5).

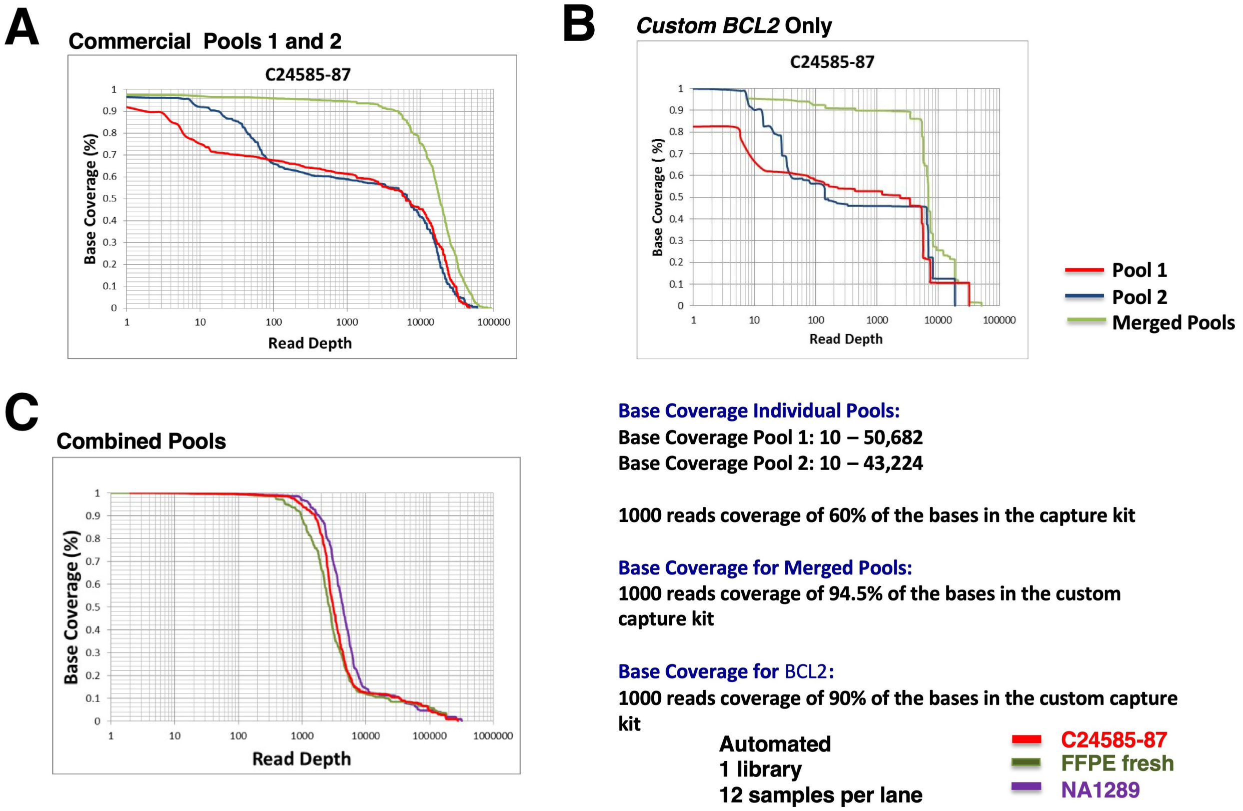

Figure S2. Base coverage across 3 pools studied.

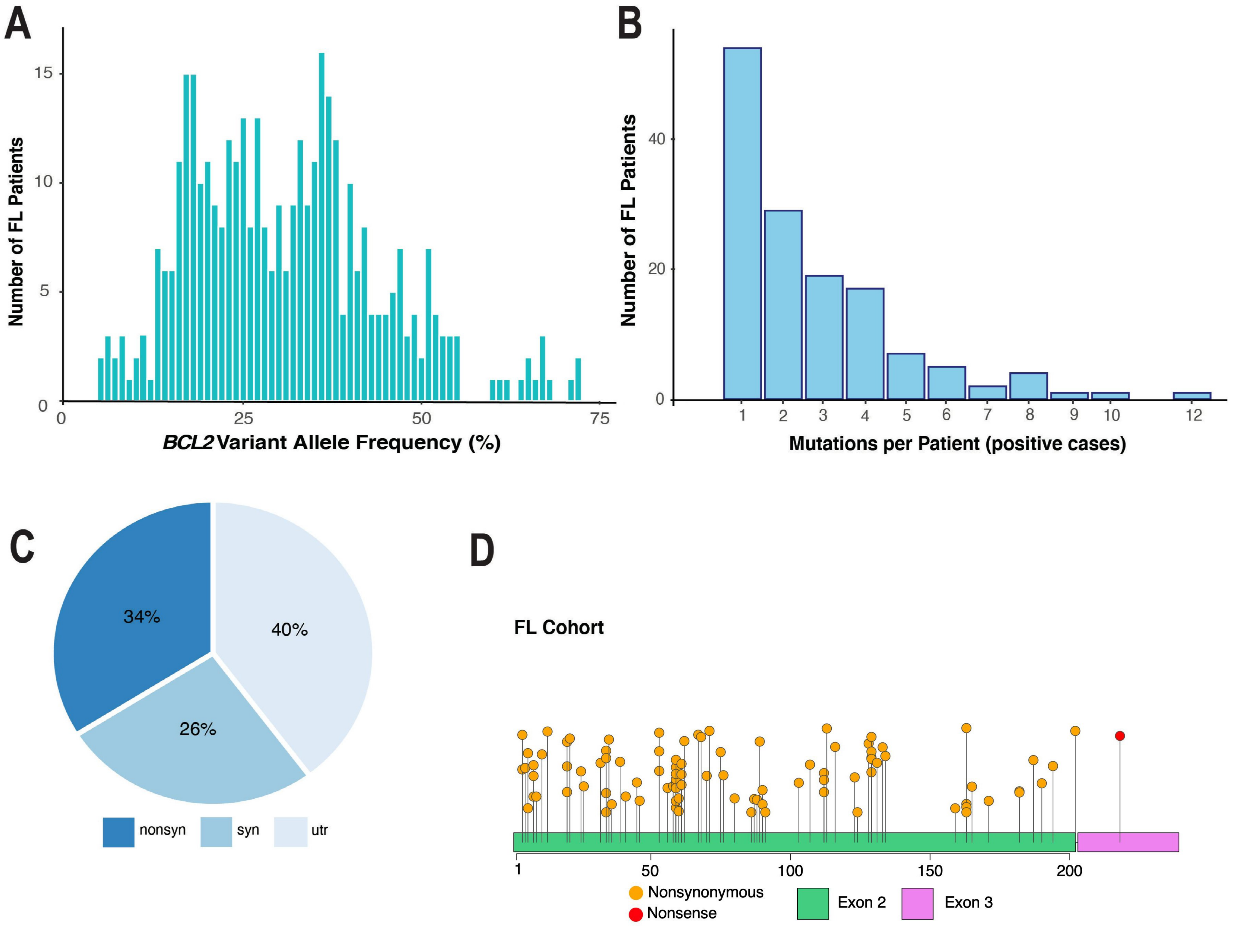

Figure S3. **Further assessment of *BCL2* mutations in present FL cohort (related to Fig. 1).** **A** Distribution of variant allele frequency across the FL cohort. **B** Number of mutations per FL patient using VAF 5% as a cut-off. **C** Mutation types using VAF 5% as a cut-off. **D** Distribution of SNVs along the *BCL2* coding exon 2 (green) and exon 3 (purple) using VAF ^{3}5% as a cut-off. Dots represent missense (orange) and nonsense (red) SNVs. Corresponding values using VAF ≥20% as a cut-off are shown in Figs. 1C and 1D.

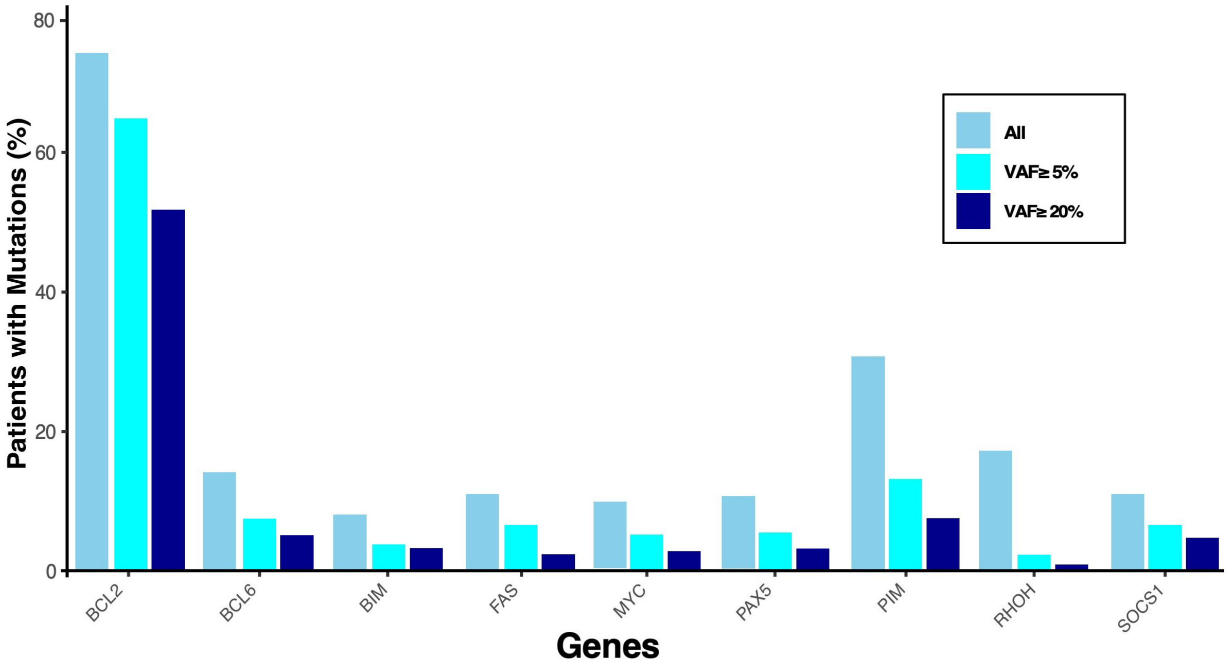

Figure S4. Percentage of FLs with somatic SNVs across AICDA target genes. Shown are percentages of cases with mutations in *BCL2*, *BCL6*, *BIM*, *FAS*, *MYC*, *PIM*, *PAX5*, *RHOH*, *SOCS1* for all detected mutations, VAF ≥5%**,** and VAF ≥20%.

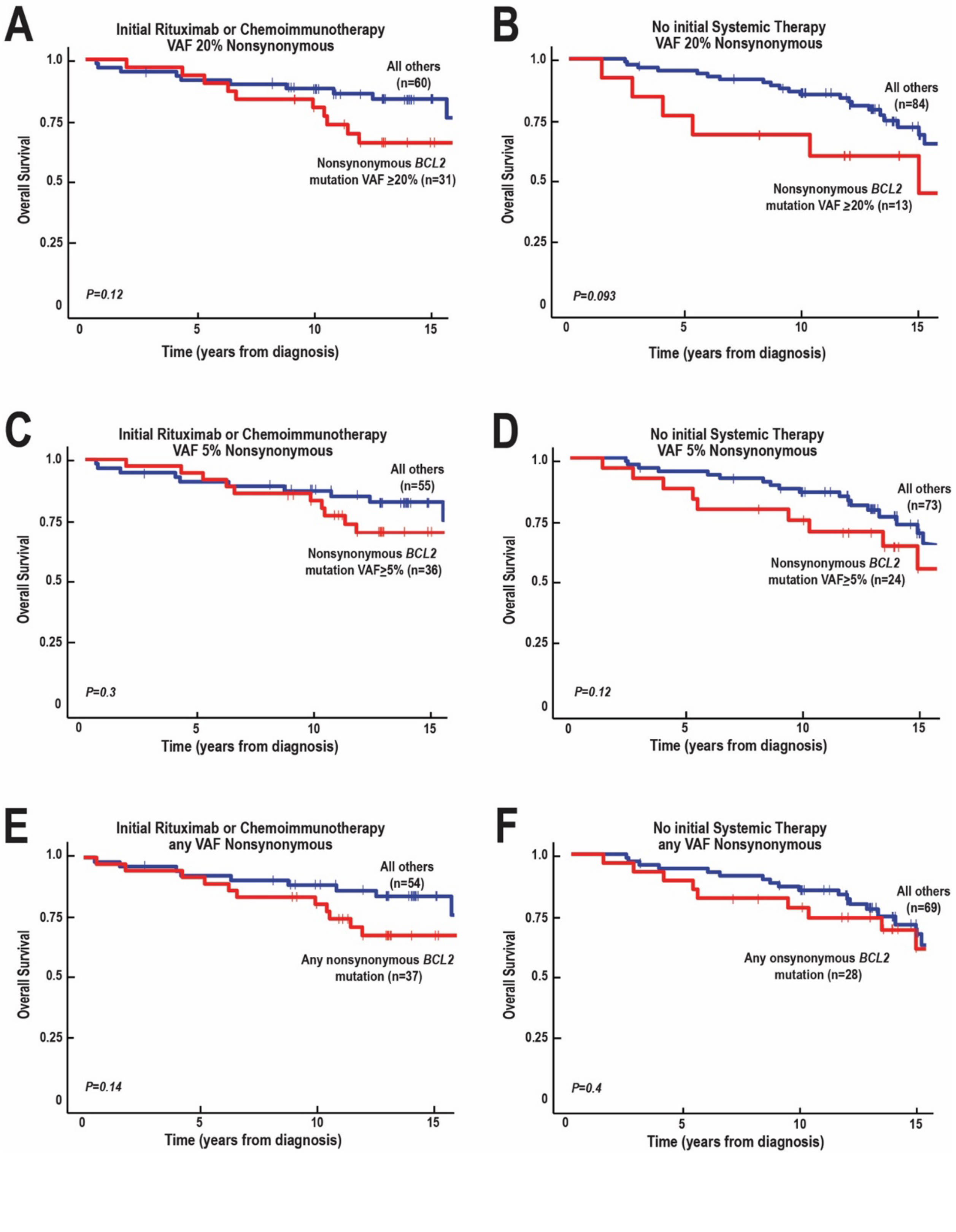
A
Initial Rituximab or Chemoimmunotherapy
VAF 20% Nonsynonymous
All others
(n=60)
Nonsynonymous BCL2
mutation VAF ≥20% (n=31)
P=0.12
Overall Survival
Time (years from diagnosis)
B
No initial Systemic Therapy
VAF 20% Nonsynonymous
All others
(n=84)
Nonsynonymous BCL2
mutation VAF ≥20% (n=13)
P=0.093
C
Initial Rituximab or Chemoimmunotherapy
VAF 5% Nonsynonymous
All others
(n=55)
Nonsynonymous BCL2
mutation VAF≥5% (n=36)
P=0.3
D
No initial Systemic Therapy
VAF 5% Nonsynonymous
All others
(n=73)
Nonsynonymous BCL2
mutation VAF≥5% (n=24)
P=0.12
E
Initial Rituximab or Chemoimmunotherapy
any VAF Nonsynonymous
All others
(n=54)
Any nonsynonymous BCL2
mutation (n=37)
P=0.14
F
No initial Systemic Therapy
any VAF Nonsynonymous
All others
(n=69)
Any onsynonymous BCL2
mutation (n=28)
P=0.4

Figure S5. Association of *BCL2* mutations detected at diagnosis with survival and risk of transformation (related to Figures 2 and 3). **A-F** Kaplan-Meier plots showing impact of *BCL2* nonsynonymous mutations with VAF ≥20% **(A, B)**, VAF ≥5% **(C, D)** or any VAF **(E, F)** on overall survival for FL patients initially treated with rituximab monotherapy or R-CHOP **(A, C, E)** and patients who were initially observed or treated with localized radiation **(B, D, F)**.
